# Priorities for child and adolescent health in Europe and Central Asia: insights to inform regional strategy from a multi-stakeholder survey

**DOI:** 10.7189/jogh.15.04306

**Published:** 2025-10-10

**Authors:** Sophie Jullien, Amy Jane Stevens, Ivelina Borisova, Susanne Carai, Gabriele Fontana, Joseph Hancock, Aleksandra Jovic, Martin W Weber, Natasha Azzopardi Muscat

**Affiliations:** 1 WHO Office for Quality of Care and Patient Safety, WHO Regional Office for Europe, Athens, Greece; 2 Bevan Community Benefit Society, Bradford, UK; 3 UNICEF Europe and Central Asia Regional Office, Geneva, Switzerland; 4 Department of Health Promotion and Development, University of Bergen, Bergen, Norway; 5 WHO Regional Office for Europe, Copenhagen, Denmark

## Abstract

**Background:**

Childhood and adolescence (0–19 years) is a critical time for laying the foundations for life-long good health and well-being. To support the development of the new World Health Organization (WHO) and the United Nations Children’s Fund (UNICEF) European Regional Strategy for Child and Adolescent Health and Well-being, we undertook a European Region-wide survey of representatives of Member States (MS), child health professionals (CHPs), and adolescents to determine priority areas for action.

**Methods:**

Stakeholders participated via structured online questionnaires in which they rated a series of problem statements concerning child and adolescent health and well-being using a five-point scale of concern. We performed descriptive analyses and calculated a regional average rating of concern for each problem statement by stakeholder group to enable them to be ranked.

**Results:**

We received responses from 40 MS representatives, 204 CHPs, and 2789 adolescents. The burden of mental health, access to mental health services, excessive screen time, problematic social media use and tobacco and nicotine product use emerged as top ten priorities across all three groups. Adolescents expressed a distinct set of priorities that nonetheless align with broader concerns, such as barriers to health care access and violence against children, including the perceived failure of health professionals to identify and act on such cases in time. Concern ratings for some problem statements varied significantly between countries, highlighting the diversity of challenges across the Region. We presented the survey findings during MS consultations to inform strategy development and ensure the content reflected the perspectives of all stakeholders.

**Conclusions:**

The inclusive consultation approach to strategy development ensured that MS priorities, the expertise of CHPs, and the voices of adolescents themselves enter into the core of ambitions to achieve ‘a healthy start for a healthy life’.

Childhood and adolescence (0–19 years) is a critical time for laying the foundations for life-long good health and well-being. Over recent decades, the World Health Organization (WHO) European Region has experienced substantial progress in child and adolescent health. However, improvement in outcomes has begun to stagnate, and significant inequalities in morbidity and mortality persist [1,2]. Children are growing up in economic, climate, geopolitical and humanitarian crises. The harmful influences of commercial determinants of health are increasing, the damage caused by the COVID-19 pandemic and its associated control measures remains evident, the growin use of social media poses an emerging health threat, and health systems across the Region are experiencing increasing organisational and resource-related pressures.

Improving the health of children and adolescents is a cross-sectoral challenge, and the development of national strategies is an established way to coordinate actions in this context. A Regional framework may be a valuable tool to guide countries and facilitate inter-country learning on successful approaches. The 2005 and 2014 WHO European strategies for child and adolescent health reflected a strong regional commitment to investing in children and adolescents [3,4]. However, simultaneous emerging and ongoing crises have increasingly drawn political focus and resources away from child and adolescent health [5]. To support continued efforts, the WHO has partnered with the United Nations Children’s Fund (UNICEF) to develop and implement a joint strategy for child and adolescent health and well-being for the WHO European Region (2026–30): ‘A healthy start for a healthy life’ [6]. To inform this strategy, we surveyed official representatives of Member States (MS), child health professionals (CHPs) and adolescents from across the Region on behalf of the WHO and UNICEF to identify priorities for action. Here we present the survey findings, with a focus on the top priority areas identified by stakeholder groups.

## METHODS

### Survey designs and study cohort

We developed a survey tailored for MS representatives and CHPs in English and Russian, in line with WHO requirements. It included 45 problem statements covering key areas of concern for child and adolescent health and well-being in the Region (**Box 1**). These were primarily informed by the WHO 2021 ‘Child and adolescent health in Europe: report on progress to 2021’ and further refined through technical input from WHO/Europe and UNICEF, reflecting emerging challenges [2].

Box 1 **Problem statements included in survey of official MS representatives and child health professionals**

Children are exposed to the aggressive promotion of alcohol, tobacco and nicotine products.

Too many women drink alcohol during pregnancy.

Asthma in children is poorly recognised and managed.

Adolescents under the age of 18 do not have access to contraception without parental or legal guardian consent.

Adolescents face barriers in accessing health services.

Adolescents under the age of 18 do not have access to mental health services without parental/legal guardian consent.

The burden of mental health problems in children and adolescents is increasing.

Children with cancer do not receive the care they need.

More children are living in poverty and experiencing food and housing insecurity.

Key data on child epidemiology are not adequately collected and reported.

Good-quality and affordable early childhood learning facilities are not accessible for many toddlers.

Rates of early initiation of breastfeeding are low.

Early intervention for children at risk of or with developmental difficulties is fragmented and scarce.

Children and adolescents spend too much time in front of screens (*e.g.* television, phone, computer, tablet).

Exclusive breastfeeding rates are low.

Among school-aged children, health and education inequalities caused by COVID-19 control measures have increased and are insufficiently addressed.

The health and well-being needs of refugee and migrant children are not being met.

Schools do not provide a health-promoting environment.

The health system lacks sufficient agility in identifying cases of violence against children and making timely referrals.

Children are not adequately taught in schools about their present and future health.

Most services are developed without the engagement or participation of children and adolescents.

Child and adolescent health services are not sufficiently integrated with social welfare and education systems to address their comprehensive health and well-being needs.

Children and adolescents are not addressed in national tuberculosis guidelines.

Most primary health care providers are not trained to care for adolescents.

Children at risk of or with developmental difficulties are identified late.

Children are left behind when their parents work abroad.

The marketing of unhealthy foods to children is aggressive.

School health services do not provide high-quality mental health support.

Neonatal or child mortality is increasing or stagnating above the lowest achievable level.

Out-of-pocket payments for children’s and adolescents’ health care are substantial.

The number of children living with overweight or obesity is increasing.

Parental counselling on child health, nutrition and development is rarely provided or of poor quality.

Parents and carers face difficulties combining work with informal caring duties.

Parents are not provided with adequate parenting skills education.

Postpartum depression in young mothers is poorly managed.

Problematic social media use among adolescents is on the rise.

Rates of road traffic injuries among children and adolescents are high.

Safe communities and neighbourhoods where children can play, engage in social and physical activities, and feel safe at home are needed.

Tobacco use among adolescents remains high, and the use of nicotine products is increasing.

Tooth decay rates among children and adolescents are high.

There are no specific arrangements to enable a smooth transition between child/adolescent and adult health care systems.

Children and adolescents are prescribed too many unnecessary antibiotics.

Children and adolescents are too often unnecessarily admitted to hospitals.

Vaccination coverage among children and adolescents is low or decreasing.

Violence against children is a major public health issue.

To enable adolescent participation, the problem statements were adapted independently by the WHO and UNICEF and piloted by their respective youth networks, leading to two adolescent surveys with a combined total of 32 statements, of which 22 were common to both (Table S1 in the **Online Supplementary Document**). The adolescent survey was available in eight languages.

Survey respondents were asked to rate the problem statements on a five-point scale of concern (1 = not a concern, 5 = high priority concern), and were given an opportunity to leave free text responses on additional priorities.

### Data collection and statistical analysis

We collected survey responses from October 2024 to January 2025 using structured online questionnaires developed and distributed *via* Microsoft Forms. The survey was shared with MS representatives (child and adolescent health focal points as nominated by the Ministries of Health) of the 53 MS of the WHO European Region *via* an email participation link. They were encouraged to consult with relevant persons across ministries to ensure responses were representative of the MS, rather than to share their individual opinion. The same survey was shared with CHPs across the Region *via* professional groups and networks, including the European Academy of Paediatrics, the European Paediatric Association, the European Confederation of Primary Care Paediatricians, and the Health Behaviour in School-aged Children network. The questionnaire was distributed by the WHO Regional Office for Europe’s Child and Adolescent Health team to focal points within these institutions, who then disseminated it through their respective channels. The youth-friendly versions of the survey were distributed to adolescents aged 12–19 years *via* WHO and UNICEF youth networks. For both CHPs and adolescents, the survey was open to all individuals within these networks who wished to respond.

We conducted this survey as part of formal collaboration with MS in the WHO European Region. Dissemination of the survey followed the standards and protocols of professional and youth networks involved (internal documents), while data were collected through a secure online form, with no personal identifiers or sensitive information recorded.

We performed descriptive analyses using Microsoft Excel, version 2410 (Microsoft Corporation, Redmond, Washington, USA) and Tableau Public, version 2024.3.1 (Salesforce, San Francisco, California, USA). Results are presented through dumbbell charts, maps and figures. We calculated the Regional average rating of priority for all the problem statements by stakeholder group. Where a rating for a problem statement was missing for a country, that country was discounted from the denominator in the calculation of the regional average. Where there was more than one response per country, as was the case in the CHP and adolescent surveys, we calculated the Regional average from average country ratings. This prevented responses from well-represented countries from skewing the regional rating. We used the Regional ratings to determine the priority ranking of problems for each stakeholder group. We otherwise reviewed the free text responses and identified common themes.

## RESULTS

**Figure 1:**
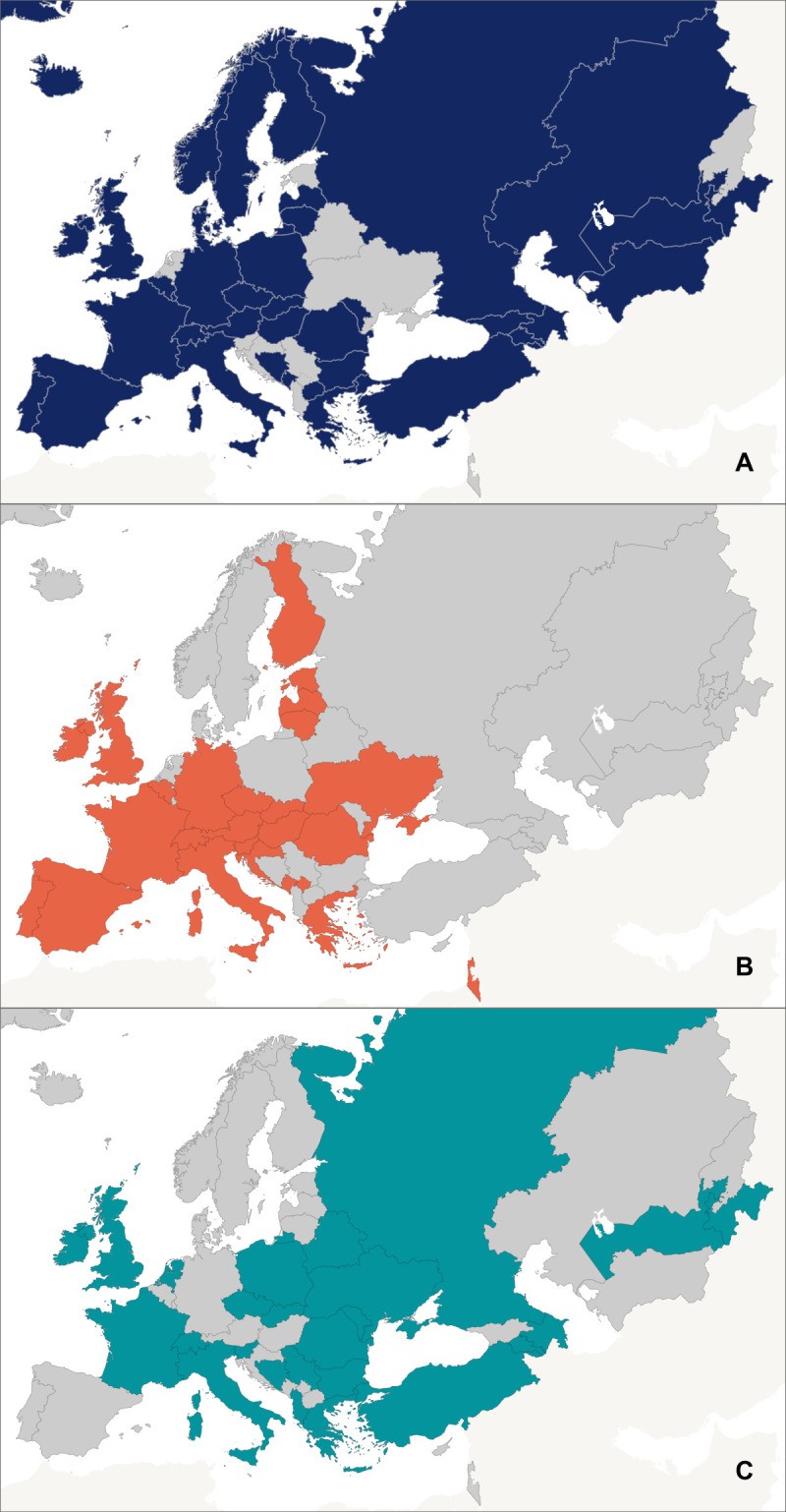
Countries providing responses to the survey by stakeholder group. **Panel A.** MS representatives. **Panel B.** Child health professionals. **Panel C.** Adolescents.

Forty MS and 204 CHPs responded to the survey. Of the 26 countries and territories represented in CHP responses, 62% only had responses from one or two CHPS in their country/territory. Ukraine had 89 responses from CHPs, Spain had 43, the UK had 13, and Greece had 11. Most respondents (75%) were paediatricians, but there were also individuals from other clinical specialties (12%), researchers (5%), children’s ombudsperson services (3%), public health (1%), dentistry (1%), and allied health professions (1%). The majority of respondents worked in primary care settings (58%), followed by hospitals (14%), university/academic institutions (12%), and specialist community clinics (5%). Responses were received from 2789 adolescents in 25 countries; 30% were from Slovakia, 28% from Uzbekistan, 9% from Romania, and 8% from Republic of Moldova (**Figure 1**).

### Priorities

**Figure 2:**
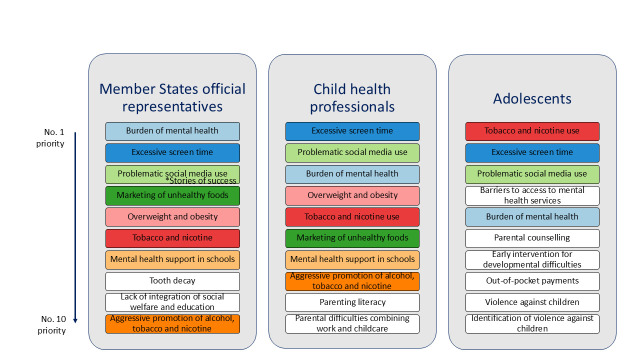
Matching priorities (colour-coded) in Regional top 10 child and adolescent health and well-being priorities, by stakeholder group

Excessive screen time, problematic social media use, mental health problems, access to mental health services, and tobacco and nicotine product use were top ten priorities for MS representatives, CHPs, and adolescents (**Figure 2**). Adolescents were presented with fewer problem statements than MS representatives and CHPs, but these did include all those that feature in the MS representative and CHP top ten priorities, except for the statement related to parental difficulties combining work and child care.

The increasing number of children living with overweight or obesity and aggressive marketing of unhealthy foods to children were top ten priorities for MS representatives and CHPs. They ranked 11th and 16th, respectively, in adolescent-rated responses. There was agreement that better integration of health with social welfare and education systems is a priority need, as it was listed 9th, 13th, and 14th in MS representatives’, CHPs’, and adolescents’ rankings, respectively. Tooth decay rates ranked 8th in MS representatives’ concerns, but were less of a priority for CHPs and adolescents, ranking 23rd and 21st, respectively.

A top ten priority for CHPs was the need for improved parenting literacy (provision of education or advice to parents on how to care for their child(ren)), a problem ranked 19th and 20th by MS representatives and adolescents, respectively. A more pressing issue from the perspective of adolescents was the need for improved parental counselling on child health, healthy eating, and development, which they ranked 6th overall. This is in contrast with MS representatives and CHPs, who ranked it 28th and 33rd, respectively. The difficulties faced by parents and carers in combining work with parenting and childcare was recognised by MS representatives and CHPs, who ranked them 17th and 10th, respectively.

Service access barriers were top adolescent priorities, but were perceived to be less of a priority by MS representatives and CHPs, though variation across the Region was evident. Inadequate early intervention for children with developmental difficulties was ranked 6th by adolescents, and 13th and 15th by MS representatives and CHPs, respectively.

Violence against children and the perceived failure of health professionals to identify and take timely action on this both featured in the top ten priorities for adolescents. These statements were ranked 12th and 15th by MS representatives and 17th and 22nd by CHPs, respectively.

Free text responses identified other shared priorities between at least two stakeholder groups including: workforce challenges; waiting lists for specialist services; bullying; sleep problems; alcohol and substance misuse; gambling; loneliness; and the need for reproductive health and sex education. Additional priorities highlighted by adolescents included: adults not understanding or responding to their needs, particularly in relation to mental health; the stigma and taboo around mental health; self-harm and suicidal ideation; school related stress and academic pressure; poor parent-child relationships; discrimination; gender-based violence; a disregard for child rights; and air pollution.

**Figure 3:**
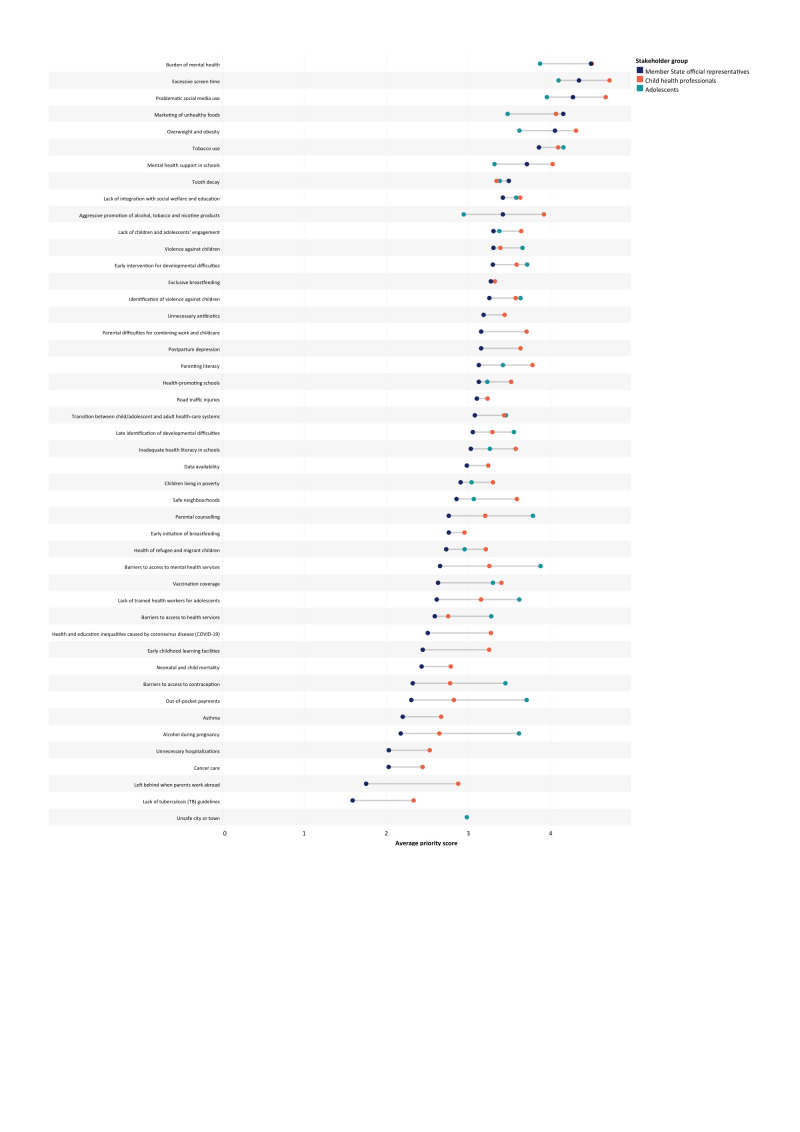
Stakeholder perspectives on child and adolescent health and well-being priorities in the WHO European Region.

The MS representatives, on average, reported lower levels of concern across all problem statements than CHPs and adolescents, with few exceptions (**Figure 3**).

### Regional diversity

**Figure 4:**
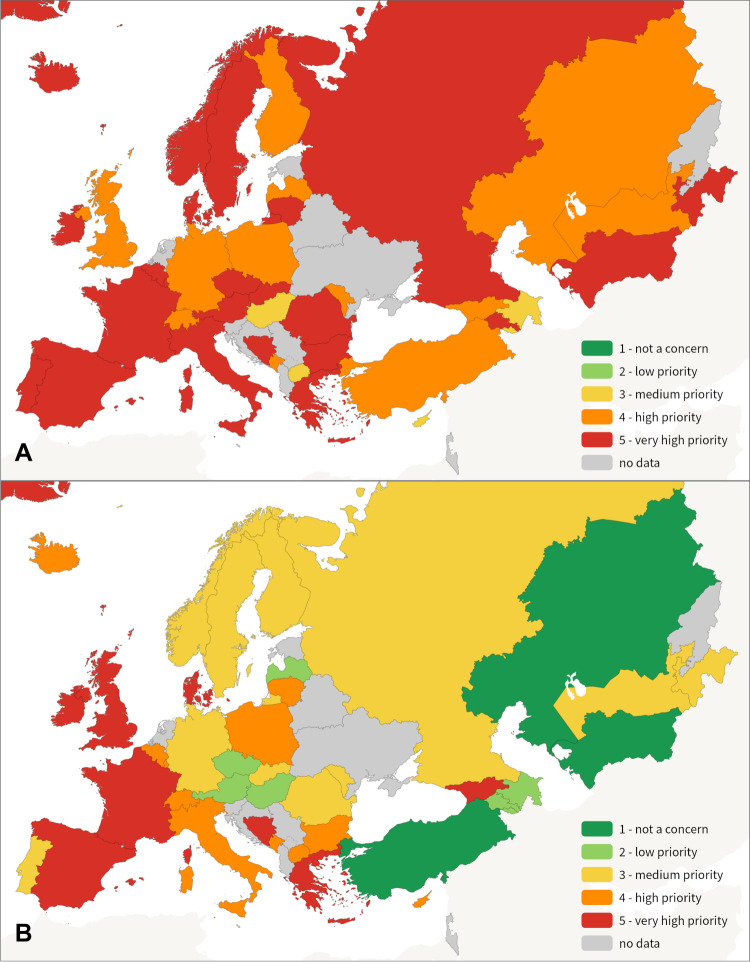
Variation in level of priority between MS representatives for the first (**Panel A**) and tenth (**Panel B**) top 10 priorities. **Panel A.** MS ratings for: ‘The burden of mental health problems in children and adolescents is increasing.’ **Panel B.** Member State ratings for: ‘Children are exposed to the aggressive promotion of alcohol, tobacco and nicotine.’

While there was clear consensus across the Region on the level of priority for some problem statements, the diversity of its challenges was reflected in responses to others (**Figure 4**).

Each of the 45 problem statements was considered a high priority concern for at least one of the 40 responding MS representatives, highlighting the breadth of needs across the Region. Priority ratings for some problem statements varied significantly between countries. For example, concern about the health and well-being needs of refugee and migrant children not being met only ranked 30th and 32nd in the 45 areas of concern based on Regional average ratings by MS representatives and CHPs, respectively. However, 28% of countries represented by MS representatives and 31% of countries represented by CHPs had a rating of 4 or more on the five-point scale of concern, demonstrating that the health of this population is a high-priority issue in some parts of the Region. Identified areas of significant priority were occasionally unique to a country: for example, free text responses from CHPs in Ukraine frequently highlighted the impacts of war and displacement on child health.

## DISCUSSION

### Inclusive consultation

Meaningful consultation is essential for developing strategies that are effective, relevant, and responsive to the needs and priorities of those affected by said strategies. Broad engagement helps ensure equity and inclusiveness, reducing decision-making bias. Top-down approaches are increasingly being replaced by purposeful stakeholder engagement, public consultations, targeted surveys, workshops, and feedback mechanisms. The findings from this survey contribute to the growing body of insights and evidence obtained from previous consultations with MS representatives, CHPs, and children and adolescents undertaken since the development of the 2005 WHO European Region strategy for child and adolescent health and development.

The survey findings highlight the necessity of involving stakeholders beyond MS representatives in strategy development to avoid blind spots in understanding the depth and breadth of child and adolescent health and well-being. Responses showed that CHPs – those working closest to the end-users of the health system – consistently perceive the child and adolescent health challenges as more severe than representatives from ministries of health, who are typically involved in system design and governance. Adolescents, meanwhile, expressed a distinct set of priorities that nonetheless align with broader concerns. For example, while they identified the lack of access to mental health services without parental or legal guardian consent as a key barrier, policymakers tended to focus on mental health as a general priority, without specifically acknowledging the unique challenges adolescents face in accessing appropriate and timely support. Similarly, adolescents highlighted the substantial out-of-pocket payments for children’s and adolescents’ healthcare as a more pressing issue than policymakers did, pointing to financial barriers that can significantly hinder access to essential services. In addition, if adolescents express strong concern about violence against children and the health sector’s limited capacity to identify and support those affected – while these issues do not feature among the top priorities of governance and service providers – this highlights a critical disconnect. It shows that greater alignment with the lived experiences and expressed needs of adolescents is essential to ensure responsive and accountable health systems. The voice of children remains largely absent in strategy development, despite their right under the United Nations Convention on the Rights of the Child to express their views in decisions that affect them, and the availability of guidance to help realise this right [7,8]. Involving children and adolescents in decision-making processes results in more effective, sustainable, and impactful outcomes [7].

The survey responses were presented to support reflection and discussion during strategy development consultations with MS representatives. Insights gathered through focus group discussions with adolescents were shared by two adolescent representatives during a consultation meeting. This provided sensemaking opportunities, garnered additional insights, and redressed power imbalances between policymakers, providers, and the recipient population by enabling all voices to be integrated into decision-making. The process facilitated iterative improvement of strategy development and ensured the content was relevant to all stakeholders.

The regional variation in the prioritisation of some child and adolescent health issues, as reflected in the survey findings, highlights the importance of ensuring that participants in global health consultation consultations represent all countries the strategy aims to serve. Failure to do so could increase health inequalities experienced between countries, as high priorities for some MS may otherwise fall off the Regional agenda. The finding that each problem statement was considered a high priority by at least one responding MS representative informed the development of a broad and comprehensive Regional strategy.

### Top priority areas

The top priorities identified are consistent with other stakeholder surveys conducted in the Region and are justified by data. This increases confidence in the reliability of the survey findings and the consequent decision to place the addressing of these issues at the forefront of the new strategy.

The burden of mental health was a priority across stakeholder groups, which aligns with the findings of other surveys [2,9,10]. Nine million adolescents aged 10–19 years in Europe live with a mental disorder, while suicide is the second most common cause of death among adolescents aged 15–19 years [11]. Half of all mental health problems in adulthood have their onset during or before adolescence [12]. The early years are essential for robust mental health. Relationships are vital in promoting well-being and mitigating against the health harms of toxic stress triggered by adverse childhood experiences [13]. Children thrive if they receive nurturing, loving, and responsive care from parents, caregivers, or other significant adults in their lives [14]. The CHPs recognised parenting skills education as a priority to be addressed (ranked 9th), but this was ranked lower (19th) by MS representative, suggesting a greater understanding and focus on this is needed at a policy-making level.

The requirement of parental consent to access mental health services ranked 4th by adolescents and 31st by MS representatives. Easy access to free mental health information and services has been identified as an urgent need by children and adolescents in other Regional surveys [15,16]. It is crucial that policymakers and service providers recognise and address the barriers children and adolescents face in accessing support. Lack of quality mental health support in schools was ranked 7th in MS representatives’ and CHPs’ concerns. The role of education settings in promoting and supporting child and adolescent mental health and well-being is being increasingly recognised [17,18]. Adolescent free text responses highlighted school-related stress and academic pressures as drivers of poor mental health, which is in line with other surveys which concluded that a change in the education system and school environment is needed [15,16]. Addressing mental health demands a systems approach, as the multiple determinants of mental health necessitate responses from health, education, social protection, and justice sectors. There was agreement among survey stakeholder groups that child and adolescent health services are not sufficiently integrated with education and social welfare systems to address their comprehensive health and well-being needs. The WHO and UNICEF have a vision to make every school a health promoting school; realisation of this ambition could help achieve a systems approach to the current mental health crisis experienced by children and adolescents in the Region [18].

Excessive screen time and problematic social media use are growing health threats which featured in the top three priorities across stakeholder groups. Eleven per cent (up from 7% in 2018) of adolescents are classified as problematic social media users, and only a third of children under five years of age are meeting screen time guidance [19,20]. The intersection between screen time and social media use and other key priority stakeholder concerns – mental health, alcohol, tobacco, and nicotine use and children living with overweight and obesity – demands attention [21–24], and consensus guidance and regulatory measures are urgently required [25].

Tobacco and nicotine product use by young people was the highest concern of adolescents. A quarter of 15-year-olds in the Region have smoked tobacco in their lifetime and over 30% have used electronic cigarettes (e-cigarettes) [26]. E-cigarette use in childhood is associated with cigarette use later in life [27]. Evidence on their harm among young people is growing, while the adverse health impact of tobacco use is well established [27,28]. The increase in adolescent e-cigarette use has been attributed to targeted advertising and exposure to online environments promoting e-cigarettes [29]. Aggressive promotion of tobacco and nicotine products and alcohol features in the top ten priorities for MS representatives and CHPs. A third of countries of the WHO European Region do not have a policy, strategy, or action plan on tobacco or alcohol control [30]. Most countries regulate traditional alcohol and tobacco advertising to protect children, but only a minority regulate marketing and advertising on digital platforms [31]. Restrictions on advertising, marketing, and point-of-sale displays are needed.

Children living with overweight or obesity remain a major priority for stakeholders, as highlighted both in the 2019 MS survey and in ongoing consultations [2]. Overweight and obesity are leading causes of mortality, morbidity, and disability in the Region [32], affecting around a quarter of children and adolescents and exhibiting an increasing prevalence in many countries [33,34]. The MS representatives and CHPs acknowledged the urgent need to address aggressive marketing of unhealthy food to children, while adolescents prioritised the need to provide parents with more information about healthy eating. Upstream system-level policies inclusive of structural, fiscal, and regulatory action is urgently needed to address obesogenic environments [35].

Violence against children and the ability of the health system to identify and respond to this were top 10 priorities for adolescents. It is notable that CHPs, who are responsible for identifying and responding to violence against children, did not see these issues as high priorities. Each year, at least 55 million children in the Region experience some form of violence, including physical, sexual, emotional, and psychological violence, and 700 children are murdered [36]. Concern about gender-based violence was cited in free text responses from adolescents. An estimated 37 million girls and women across the Region have experienced sexual violence during their childhood and nearly 25% of all girls aged 15–19 years who have been in a relationship will have experienced intimate partner violence by the time they turn 20 years old [37]. Implementation and enforcement of laws to protect children from violence, a shift away from harmful gender and social norms, safe environments, parent/caregiver support, and responsive support services are needed [38].

### The need for a system-wide and multi-sector response

Many top priorities for child and adolescent health and well-being are interconnected, with some issues acting as drivers or exacerbating factors for others. For example, problematic social media use, exposure to violence, and body dissatisfaction among those living with overweight and obesity are all linked to mental health [10,39]. Shared solutions, such as regulating harmful commercial determinants, can address multiple concerns, including mental health, problematic social media use, cyberbullying, tobacco and nicotine use, and overweight and obesity. These overlaps highlight the need for a holistic, system-wide approach and intersectoral action. Collaboration across health, education, social welfare, nutrition and urban planning is essential to create healthy environments where children can thrive. Countries must utilise all levers available to improve health and well-being of children and adolescents across the Region. This includes adherence to international standards, political commitments, and adequate legislation.

### Limitations

These findings reflect the views of the survey respondents and are not necessarily an accurate representation of key priority areas across the WHO European Region as a whole; the MS representatives, CHPs or adolescents did not represent all of its countries. While representation from MS was relatively broad, adolescent responses were more concentrated, with 58% coming from just two countries – Slovakia and Uzbekistan. The availability of the surveys in a limited number of languages may have impacted the response rate in some countries and distribution networks for CHP and adolescents’ surveys may not have reached all countries. While some survey responses will have been evidence-based, others may have reflected personal, academic, or professional perspectives. Interpretation of statements may have varied due to cultural context, language, or literacy levels. The perspectives of children under 12 years of age were not collected and the survey was not adapted to meet the communication needs of adolescents with learning disabilities or those experiencing illiteracy. Adolescent survey participants are unlikely to represent the most marginalised or under-represented young people.

## CONCLUSIONS

The WHO and UNICEF strategy for child and adolescent health and well-being for the WHO European Region (2026–30) presents an opportunity to unite the Region in its commitment to ensuring all children and adolescents have the right to health, protection and development. The strategy offers direction to countries in supporting a comprehensive and integrated approach to addressing the highest priority areas. The survey findings have informed the strategy’s development, ensuring that MS priorities, the expertise of CHPs, and the voices of adolescents themselves are at the heart of ambitions to achieve ‘a healthy start for a healthy life’.

## Additional material


Online Supplementary Document
