# Supplementary material for: Priorities for child and adolescent health in Europe and Central Asia: insights to inform regional strategy from a multi-stakeholder survey
Source: J Glob Health. 2025 Oct 10;15:04306. doi: 10.7189/jogh.15.04306 (PMC12512001; doi:10.7189/jogh.15.04306)
Supplement: Online Supplementary Document [file jogh-15-04306-s001.pdf]

Supplement to: Jullien S, Stevens AJ, Borisova I, Carai S, Fontana G, Hancock J, Jovic A, Weber MW, Azzopardi Muscat N. Priorities for child and adolescent health in Europe and Central Asia. J Glob Health. 2025;15:04306.

Table S1. List of abbreviated and full survey statements by stakeholder group

Abbreviated statement for data visuals	Full statement included in survey of official Member State representatives and child health professionals	Full statement included in WHO adolescent survey	Full statement included in UNICEF adolescent survey
Aggressive promotion of alcohol, tobacco and nicotine products	Children are exposed to the aggressive promotion of alcohol, tobacco and nicotine products.	Children are shown a lot of advertisements for alcohol, cigarettes and e-cigarettes (vapes).	I am shown a lot of advertisements for alcohol, cigarettes and e-cigarettes (vapes).
Alcohol during pregnancy	Too many women drink alcohol during pregnancy.	Too many women drink alcohol during pregnancy.	
Asthma	Asthma in children is poorly recognized and managed.		
Barriers to access to contraception	Adolescents under the age of 18 do not have access to contraception without parental or legal guardian consent.	Teenagers under the age of 18 cannot get contraception without the consent (permission) of their parent/guardian.	Teenagers under the age of 18 cannot get contraception without the consent (permission) of their parent/guardian.
Barriers to access to health services	Adolescents face barriers in accessing health services.	It is difficult for teenagers to access health services.	It is difficult for teenagers to access health services.
Barriers to access to mental health services	Adolescents under the age of 18 do not have access to mental health services without parental/legal guardian consent.	Teenagers under the age of 18 cannot access mental health services without the consent (permission) of their parent/guardian.	Teenagers under the age of 18 cannot access mental health services without the consent (permission) of their parent/guardian.
Breastfeeding support			
Burden of mental health	The burden of mental health problems in children and adolescents is increasing.	The number of children with mental health problems (e.g. depression, anxiety, eating disorders) is increasing.	I am concerned about the number of children with mental health problems (e.g. depression, anxiety, eating disorders).
Cancer care	Children with cancer do not receive the care they need.		
Children living in poverty	More children are living in poverty and experiencing food and housing insecurity.	An increasing number of children are living in poverty and do not have enough money for food or good housing.	Many children in my community are living in poverty and do not have enough money for food or good housing.
Data availability	Key data on child epidemiology are not adequately collected and reported.		
Early childhood learning facilities	Good-quality and affordable early childhood learning facilities are not accessible for many toddlers.		
Early initiation of breastfeeding	Rates of early initiation of breastfeeding are low.		
Early intervention for developmental difficulties	Early intervention for children at risk of or with developmental difficulties is fragmented and scarce.	Children with developmental difficulties (e.g. difficulties with their speech, understanding, movement, learning, or managing their emotions as expected for their age) are not getting the early help they need.	
Excessive screen time	Children and adolescents spend too much time in front of screens (e.g. television, phone, computer, tablet).	Children and teenagers spend too much time in front of screens (e.g. television, phone, computer, tablet).	Children and teenagers spend too much time in front of screens (e.g. television, phone, computer, tablet).
Exclusive breastfeeding	Exclusive breastfeeding rates are low.		
Guidance for screen time			
Health and education inequalities caused by COVID-19	Among school-aged children, health and education inequalities caused by COVID-19 control measures have increased and are insufficiently addressed.		

Health of refugee and migrant children	The health and well-being needs of refugee and migrant children are not being met.	The health and well-being needs of refugee and migrant children are not being met.	
Health-promoting schools	Schools do not provide a health-promoting environment.	School settings do not encourage and support healthy behaviours in children and do not promote good well-being (e.g. limited access to healthy food and opportunities for exercise, children do not feel safe, children feel unsupported by school staff).	
Identification of violence against children	The health system lacks sufficient agility in identifying cases of violence against children and making timely referrals.	Health professionals (e.g. doctors and nurses) are too slow to identify and help children and teenagers who have experienced violence.	
Inadequate health literacy in schools	Children are not adequately taught in schools about their present and future health.	Schools do not teach children about how to look after their health and well-being.	My school does not teach me about how to look after my health and well-being.
Lack of children and adolescents' engagement	Most services are developed without the engagement or participation of children and adolescents.	Most health services are developed without involving children and teenagers or asking them their ideas about what is needed.	
Lack of integration with social welfare and education	Child and adolescent health services are not sufficiently integrated with social welfare and education systems to address their comprehensive health and well-being needs.	Health services, social services and education systems need to work together better to provide the care and support children and teenagers need.	
Lack of TB guidelines	Children and adolescents are not addressed in national tuberculosis guidelines.		
Lack of trained health workers for adolescents	Most primary health-care providers are not trained to care for adolescents.	Doctors and nurses need better training in providing health care for teenagers.	Doctors and nurses need better training in providing health care for teenagers.
Late identification of developmental difficulties	Children at risk of or with developmental difficulties are identified late.	Children who need help because of developmental difficulties (e.g. difficulties with their speech, understanding, movement, learning, or managing their emotions as expected for their age) are being identified late.	
Left behind when parents work abroad	Children are left behind when their parents work abroad.		
Marketing of unhealthy foods	The marketing of unhealthy foods to children is aggressive.	Children are shown a lot of advertisements for unhealthy foods.	I am shown a lot of advertisements for unhealthy foods.
Mental health support in schools	School health services do not provide high-quality mental health support.	Schools do not provide good mental health support.	My school does not provide good mental health support for students.
Neonatal and child mortality	Neonatal or child mortality is increasing or stagnating above the lowest achievable level.		
Out-of-pocket payments	Out-of-pocket payments for children's and adolescents' health care are substantial.	Children's health care is expensive for teenagers and their families.	Children's health care is expensive for teenagers and their families.
Overweight and obesity	The number of children living with overweight or obesity is increasing.	The number of children who are overweight is increasing.	I am concerned that the number of children who are overweight is increasing.
Parental counselling	Parental counselling on child health, nutrition and development is rarely provided or of poor quality.	Parents need more information about child health, healthy eating and child development.	Parents need more information about child health, healthy eating and child development.
Parental difficulties for combining work and childcare	Parents and carers face difficulties combining work with informal caring duties.		
Parenting literacy	Parents are not provided with adequate parenting skills education.	Parents do not get enough education or advice on how to care for their child/children.	Parents do not get enough education or advice on how to care for their child/children.
Parental mental health and well-being			
Parental support by health workers			

Polluted air, water, unsafe environments			
Postpartum depression	Postpartum depression in young mothers is poorly managed.		
Problematic social media use	Problematic social media use among adolescents is on the rise.	The number of children and teenagers addicted to social media is increasing.	I am concerned about children and teenagers being addicted to social media.
Road traffic injuries	Rates of road traffic injuries among children and adolescents are high.	Injuries in children from road traffic accidents are high.	
Safe neighbourhoods	Safe communities and neighbourhoods where children can play, engage in social and physical activities, and feel safe at home are needed.	Many children do not live in areas where they feel safe to play and have opportunities to do sports and fun activities with other teenagers.	Many young people in my community do not feel safe to play and have opportunities to do sports and fun activities with others their own age.
Tobacco use	Tobacco use among adolescents remains high, and the use of nicotine products is increasing.	A high number of teenagers are smoking cigarettes and e-cigarettes (vapes).	I am concerned about the number of teenagers smoking cigarettes and e-cigarettes (vapes).
Tooth decay	Tooth decay rates among children and adolescents are high.	There are a high number of children with tooth decay (holes in teeth which can cause pain and infections).	I am concerned about the number of children with tooth decay (holes in teeth which can cause pain and infections).
Transition between child/adolescent and adult health-care systems	There are no specific arrangements to enable a smooth transition between child/adolescent and adult health-care systems.	Moving between child and adult health services is not smooth.	Moving between child and adult health services is not smooth.
Unnecessary antibiotics	Children and adolescents are prescribed too many unnecessary antibiotics.		
Unnecessary hospitalizations	Children and adolescents are too often unnecessarily admitted to hospitals.		
Unsafe city or town			I feel the streets in my city or town are unsafe for me.
Vaccination coverage	Vaccination coverage among children and adolescents is low or decreasing.	Not enough children and teenagers are getting vaccinated against diseases.	Not enough children and teenagers are getting vaccinated against diseases.
Violence against children	Violence against children is a major public health issue.	Many children and teenagers experience violence from adults or other teenagers.	Many children and teenagers experience violence from adults or other teenagers.
